# Case report: Favipiravir-induced bluish corneal discoloration in infant with COVID-19

**DOI:** 10.3389/fped.2023.1154814

**Published:** 2023-04-19

**Authors:** Paveewan Jiravisitkul, Saraiorn Thonginnetra, Rintra Wongvisavavit

**Affiliations:** ^1^ Department of Pediatrics, Chulabhorn Hospital, Chulabhorn Royal Academy, Bangkok, Thailand; ^2^ Princess Srisavangavadhana College of Medicine, Chulabhorn Royal Academy, Bangkok, Thailand

**Keywords:** COVID-19, favipiravir, bluish discoloration, cornea, infant, case report

## Abstract

This report describes a case of a male infant diagnosed with severe acute respiratory syndrome coronavirus 2 (SARS-CoV-2) infection who was prescribed favipiravir therapy. The mother noticed a discoloration of the child's eyes within 18 h of therapy, and the cornea returned to normal color within 5 days of medication cessation. This case report highlights the need for monitoring of favipiravir therapy in children due to the potential side effect of corneal discoloration, which has not yet had its long-term effects identified.

## Introduction

Through December 2022, nearly 700 million cases of severe acute respiratory syndrome coronavirus 2 (SARS-CoV-2) infections have been confirmed and over 6.5 million people have died as a result of the coronavirus disease 2019 (COVID-19) outbreak (1). Although children often have milder conditions than adults, roughly 37% of those who are diagnosed will require hospitalization or develop severe disease (2). Currently, recommendations for managing COVID-19 in children are limited. Effective measures, such as small-molecule inhibitors (3, 4), bioactive natural products (5, 6), and traditional medicine (7) are greatly needed to against the SARS-CoV-2 infection. However, promising magic bullets still do not exist in pediatric patients. In accordance with the Thai National Treatment Guidelines for COVID-19 issued by the Ministry of Public Health 2022 (8), favipiravir is the mainstay of antiviral treatment for COVID-19 in children with mild-to-moderate illness. The most common side effects of favipiravir include mild hyperuricemia, diarrhea, and neutropenia, which together account for roughly 20% of adverse events (9). However, the first case of an unusual adverse effect of favipiravir therapy—the bluish discoloration of the cornea—was reported in 2021 by Raiturcar et al. (10). To the best of our knowledge, this adverse effect has not been reported in a pediatric patient to date. Therefore, in this case report, we aimed to share our clinical experience with bluish cornea discoloration in a pediatric patient following favipiravir treatment.

## Case presentation

**Figure 1:**
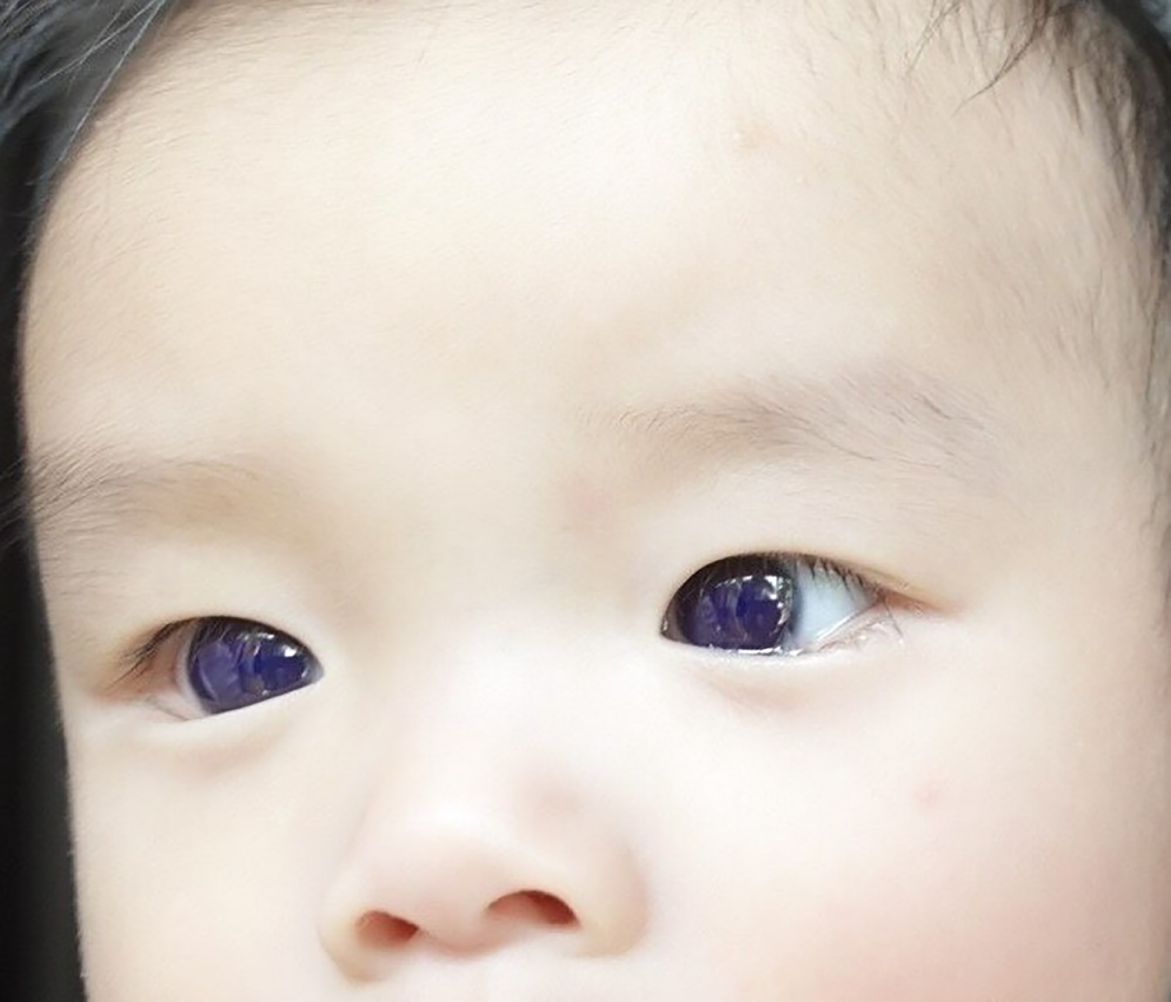
The bright blue color of the cornea at 18 h after favipiravir therapy.

**Figure 2:**
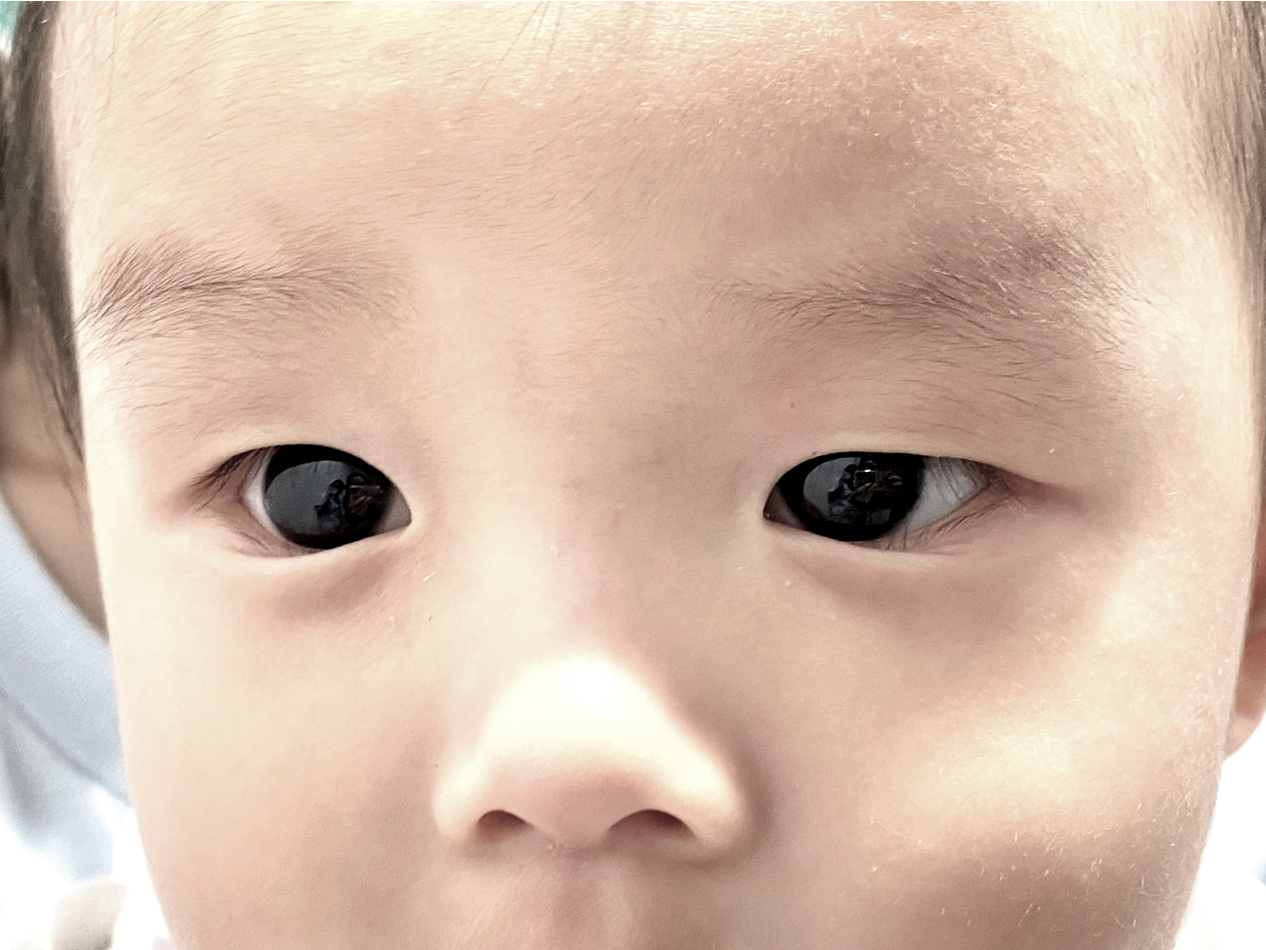
Eye examination 2 weeks after SARS-CoV-2 infection. Clear corneas without bluish discoloration are observed.

A 6-month-old male patient presented with a fever and cough lasting 1 day. The patient was diagnosed with COVID-19 using the Novel Coronavirus (COVID-19) Antigen test kit, manufactured by Zhejiang Aiminde Biotechnology in China, which yield a positive result from a nasopharyngeal swab. Favipiravir (200 mg/tab) was prescribed as first-line therapy at a dose of 82 mg/kg/day on Day 1 and favipiravir syrup (CRA Favi-Kids; 800 mg/60 ml) at a dose of 29 mg/kg/day on Days 2–5. The child's mother detected a bluish discoloration of the cornea 18 h after favipiravir therapy was initiated. The bright blue color of the cornea was observed under sunlight (Figure 1). No bluish discoloration was observed in other areas such as skin, nails, or oral and nasal mucosa. Symptoms improved after 3 days of favipiravir therapy. The pediatrician advised that the patient discontinue therapy because of favipiravir-induced corneal discoloration. The cornea returned to normal color on Day 5 after stopping the medicine. An eye examination was performed by an ophthalmologist using a handheld slit lamp 2 weeks after SARS-CoV-2 infection. The patient was able to fix and follow the light in all directions. The cornea was clear and lacked a bluish corneal hue (Figure 2). No blue pigment deposit was observed on the surface of the iris or the anterior lens capsule. The anterior chamber was deep and clear and lacked content. No evidence of fluorescence was observed when a cobalt blue filter was applied.

## Discussion

Favipiravir, a nucleotide analogue targeting the viral RNA-dependent RNA polymerase, has been approved in Japan for the treatment of patients with emerging pandemic influenza since 2014 and has also been used to treat Ebola and other viral diseases (11). In 2020, favipiravir was first used against severe acute respiratory syndrome coronavirus 2 in Wuhan, the center of the global COVID-19 outbreak (12), and it has also been received emergency use authorization in several countries against SARS-CoV-2 infection (13). A recent meta-analysis has shown that treatment with favipiravir leads to a higher rate of viral clearance and shorter clinical improvement time in hospitalized patients (14). Nonetheless, the drug did not benefit individuals who were not hospitalized (14).

Despite the positive results seen in hospitalized patients, there are several side effects associated with the use of favipiravir. Mild hyperuricemia, diarrhea, and neutropenia were the most frequently reported side effects and accounted for 20% of all adverse events (9). Furthermore, several rare adverse effects have been documented, including the bluish discoloration of the cornea (10), temporary visual blurring (15), and fluorescence of the ocular surface (15). However, these adverse reactions were observed to have resolved after discontinuation of the medication, as reported by Raiturcar et al. who documented the restoration of normal corneal color within a day (10). However, it is noteworthy that the resolution of bluish corneal discoloration was comparatively slower in our case report. Various factors, such as age, dosage, and treatment duration, may contribute to this difference. The exact reason for this delay remains unclear; however, a decrease in urine excretion could be a plausible contributing factor. In support of this possibility, a prior investigation observed a higher frequency of uric acid elevation in younger patients treated with favipiravir, which could be linked to decreased urine output (9). It is crucial to monitor and report such cases to gain a comprehensive understanding of the potential effects of medication on corneal discoloration and their resolution, particularly among children, as the incidence of this adverse event remains uncertain.

In addition to the potential for corneal discoloration, favipiravir has also been shown to cause fluorescence in human hair and nails (16). This adverse effect may be due to the drug, its metabolites, or additional tablet components such as titanium dioxide and yellow ferric oxide (10, 15). Studies have shown that the active phosphorylated metabolite of favipiravir is found in human plasma and that there is a linear correlation between its concentration and the intensity of fluorescence (16). In subsequent laboratory investigations (15), fluorescence characteristics of favipiravir tablet were examined under ultraviolet light, and the observations confirmed the fluorescence of the drug, which signified a robust association between the fluorescence of the ocular surface and favipiravir. Additionally, a direct relationship was found between the concentration of favipiravir and fluorescence intensity within a specific range (40–280 ng/ml). This method demonstrated a high level of accuracy in determining the concentration of favipiravir in pharmaceutical formulations, with a limit of detection of 9.44 ng/ml and a quantitation limit of 28.60 ng/ml (17).

Despite the potential for adverse effects, favipiravir remains the mainstay of oral antiviral treatment in Thailand for children with COVID-19. The current working dose is 35 mg/kg twice daily on Day 1 followed by 15 mg/kg twice daily on Days 2–5 (5). However, recent research has raised concerns regarding the safety and efficacy of favipiravir. The Prevent Severe COVID-19 study showed that patients with COVID-19 should not be treated with favipiravir, given its lack of demonstrated efficacy as measured by time to sustained clinical recovery, progression to severe COVID-19, and cessation of viral shedding (13).

## Conclusion

This case report highlights an unusual adverse effect of favipiravir therapy in the youngest known patient receiving the drug for the treatment of SARS-CoV-2 infection. While favipiravir is currently the mainstay of oral antiviral treatment for children with COVID-19, its safety profile in children who are still in the developmental stage is uncertain. Therefore, monitoring the long-term safety of favipiravir used in pediatric patients is of utmost importance. The reported adverse event, although rare, should be taken seriously and closely monitor in future cases. Further studies are needed to determine the incidence of these adverse effect and its potential long-term consequences on corneal health.

## Data Availability

The original contributions presented in the study are included in the article/Supplementary Material, further inquiries can be directed to the corresponding author.
